# Ras-association domain family 10 acts as a novel tumor suppressor through modulating MMP2 in hepatocarcinoma

**DOI:** 10.1038/oncsis.2016.24

**Published:** 2016-06-27

**Authors:** W Liu, J Wang, L Wang, C Qian, Y Qian, H Xuan, W Zhuo, X Li, J Yu, J Si

**Affiliations:** 1 Department of Gastroenterology, Sir Run Run Shaw Hospital, Zhejiang University, Hangzhou, China; 2 Institute of Gastroenterology, Zhejiang University, Hangzhou, China; 3 Postgraduate at Institute of Gastroenterology, Zhejiang University; The First People's Hospital of Xiaoshan, Hangzhou, China; 4 Institute of Digestive Disease and Department of Medicine and Therapeutics, State Key Laboratory of Digestive Disease, Li Ka Shing Institute of Health Sciences, Chinese University of Hong Kong, Hong Kong, China

## Abstract

Ras-Association Domain Family 10 (RASSF10) is the last identified member of the RASSF family. The functional characteristics of this new gene in human cancers remain largely unclear. Here, we examined RASSF10 for the biological functions and related molecular mechanisms in hepatocellular carcinoma (HCC). We found that RASSF10 is expressed in normal human liver tissue, but is silenced or down-regulated in 62.5% (5/8) of HCC cell lines. The mean expression level of RASSF10 was significantly lower in primary HCCs compared with their adjacent normal tissues (*P*<0.005, *n*=52). The promoter methylation contributes to the inactivation of RASSF10 as demonstrated by bisulfite genomic sequencing and demethylation treatment analyses. Transgenic expression of RASSF10 in silenced HCC cell lines suppressed cell viability, colony formation and inhibited tumor growth in nude mice (QGY7703, *P*<0.01; HepG2, *P*<0.05). Furthermore, RASSF10 was shown to induce the cell accumulation in G1 phase with the increase of p27, as well as the decrease of cyclinD1 and CDK2/CDK4. Over-expression of RASSF10 also inhibited HCC cells migration (*P*<0.01) or invasion (*P*<0.05). Adhesion genes array revealed that Matrix Metalloproteinase 2 (MMP2) was a downstream effector of RASSF10. RASSF10 acting as a tumor suppressor to inhibit HCC invasion partially mediated by Focal Adhesion Kinase or p38 MAPK to decrease the accumulation of MMP2. Our study suggests that RASSF10 acts as a tumor suppressor for HCC.

## Introduction

Hepatocellular carcinoma (HCC) remains the fifth most frequent cancer in the world and has a poor prognosis in the advanced stage.^1^ It has been recognized that inactivation of tumor suppressor genes by epigenetic events, including promoter CpG islands hypermethylation, is an important mechanism during HCC initiation and progression.^2, 3, 4^ Demethylation agents have been introduced into several malignant diseases such as lung cancer,^5, 6, 7^ so investigations of novel genes silenced by methylation would provide more insights into the molecular mechanisms and strategies of treatment for tumorigenesis.

The Ras-Association Domain Family (RASSF) is a group of proteins containing ten family members. RASSFs including RASSF1A, RASSF2, RASSF4, RASSF5, RASSF6 and RASSF8 have been implicated as tumor suppressors in various kinds of cancers by interactions with Ras GTPases, modulation of Hippo pathway, apoptosis and cell cycle.^8, 9, 10, 11^ Furthermore, the distinct inactivation of RASSF1 or RASSF2 transcripts has been indicated as a result of CpG island promoter hypermethylation.^12, 13, 14, 15^ RASSF10, the latest member of the RASSF family, has been frequently inactivated by aberrant promoter hypermethylation in several human cancers, including thyroid cancer, lung cancer and gastric carcinoma.^16, 17, 18, 19, 20, 21, 22, 23, 24^ RASSF10 is implicated to induce lung cancer cell apoptosis^22^ and repressed gastric carcinoma growth through inhibition of Wnt/β-catenin signaling pathway.^24^ However, the functional role and the molecular mechanisms of RASSF10 in carcinogenesis of HCC remain largely unclear.

In the present study, we investigated the expression profile of RASSF10 in HCC cells and discovered the frequent loss of RASSF10 expression due to promoter methylation in HCC. Further functional studies revealed that RASSF10 resulted in significant suppression of HCC invasion or migration by inhibition of MMP2, which is mediated by FAK signaling and p38 MAPK pathway. Our results support RASSF10 could function as a novel suppressor in HCC.

## Results

### Frequent down-regulation or silence of RASSF10 in HCC

**Figure 1:**
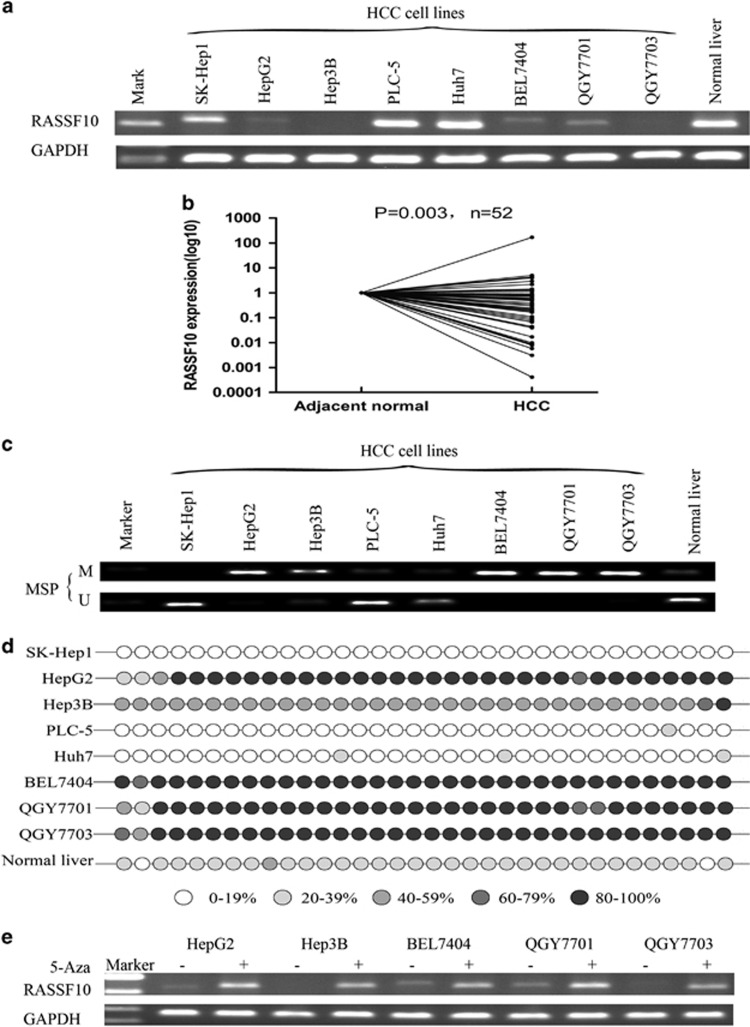
The expression of RASSF10 and its promoter methylation status. (**a**) The expression profile of RASSF10 mRNA in HCC cell lines and normal liver tissue by RT-PCR. (**b**)The mRNA expression level of RASSF10 was significantly down-regulated in primary HCCs as compared with their adjacent normal tissues by quantitative real-time PCR (*P*=0.003, *n*=52). RASSF10 expression level was normalized with the GAPDH mRNA level. (**c**) Methylation-specific polymerase chain reaction showed methylation of RASSF10 in HCC cell lines. M, methylated DNA; U, unmethylated DNA. (**d**) BGS confirmed the methylation status of RASSF10 in HCC cell lines and normal control. (**e**) RASSF10 mRNA expression was restored following 5-aza-DC treatment. GAPDH was used as a control for equal loading.

We initially examined the expression of RASSF10 in a panel of eight HCC cell lines and found down-regulated or silenced expression of RASSF10 in 62.5% (5/8) of HCC cell lines but detected in normal human liver tissue (Figure 1a). To further investigate the expression pattern of RASSF10 in primary HCCs, we have collected 52 paired surgery samples (44 male and 8 female with a mean age of 58 years) for RASSF10 mRNA evaluation in this study. In the total of 52 paired samples, the mRNA expression level of RASSF10 was significantly down-regulated in primary HCCs as compared with their adjacent normal tissues (*P*=0.003, *n*=52) (Figure 1b), indicating an aberrant gene silencing of RASSF10 in HCC.

### Promoter hypermethylation contributes to RASSF10 gene silencing

We next investigated the methylation status of RASSF10 promoter in HCC by methylation-specific polymerase chain reaction. Full or partial methylation was observed in HCC cell lines (BEL7404, QGY7701, QGY7703, HepG2, and Hep3B), showing the silenced or down-regulated expression of RASSF10 (Figure 1c).

The methylaion density within the promoter region was then characterized by bisulfite genomicsequencing (BGS), which validated a dense methylation in those HCC cell lines with silenced or down-regulated RASSF10, but much milder methylation levels in normal liver (Figure 1d, Supplementary Figure 1a).

To confirm whether the promoter methylation is involved in the silencing of RASSF10, five HCC cell lines (BEL7404, QGY7701, QGY7703, HepG2 and Hep3B) with silenced RASSF10 were treated with 5-Aza-DC for 72 h, which restored RASSF10 expression in all of them (Figure 1e).

### Suppression of cell proliferation by RASSF10 in HCC cells

**Figure 2:**
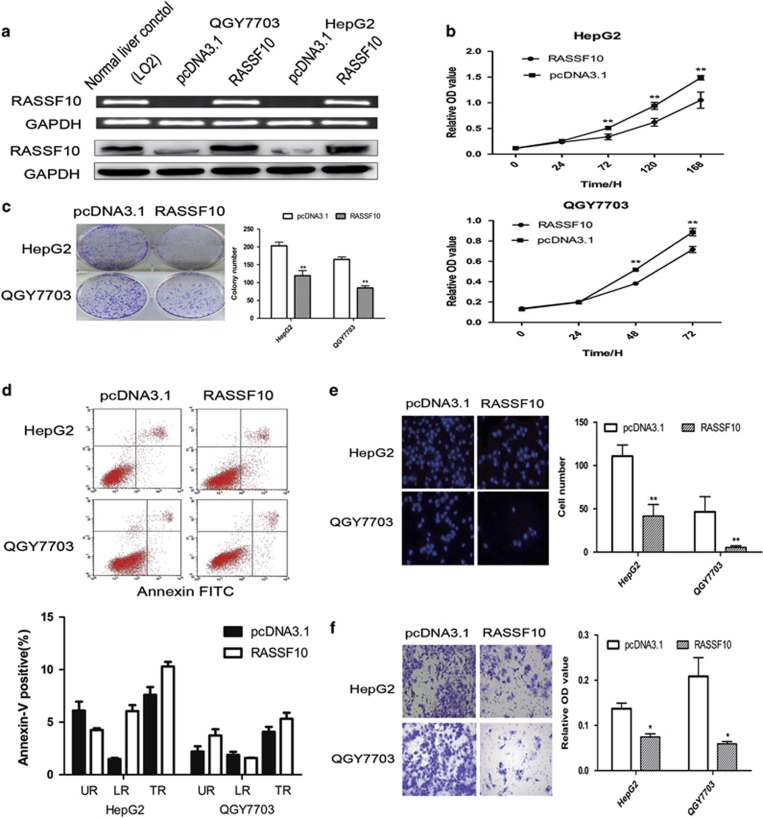
RASSF10 inhibits HCC cell growth and cell invasion. (**a**) Restored expression of RASSF10 in QGY7703 and HepG2 cell lines was evidenced by RT-PCR and western blot. As compared with the normal liver control, no special higher levels of RASSF10 were observed in RASSF10-transfected HCC cells. GAPDH was used as internal controls. (**b**) RASSF10 significantly inhibited cell viability in HCC cell lines. (**c**) The monolayer colony formation assays. Left panel shows the representative images of the colony formation in HCC cell lines transfection with pcDNA3.1/RASSF10 or empty vector (pcDNA3.1). Quantitative analysis of colony numbers is shown in the right panel. (**d**) Effects of re-expression of RASSF10 on HCC cells apoptosis in HepG2 and QGY7703 cells. Apoptosis was not influenced by the restored expression of RASSF10, which was confirmed by FITC Annexin V Apoptosis Detection assay. Representative results of cell apoptosis were showed. (**e**) Cell migration assay was performed in modified Boyden transwell chambers assay. The migratory cells stained with DAPI display on the left panel. The mean number of visible cells was counted by fluorescence microscope in five random high power fields. (**f**) Cell invasion assay was used to assess cell invasion. Invaded cells were stained with cell stain solution, and then detected on a standard microplate reader (560 nm). All data represent the average of three independent experiments in duplicate. Data are mean±s.d. The asterisk indicates statistical significance (*P<0.05, **P<0.01).

To determine whether RASSF10 acts as a tumor suppressor in HCC, we thus examined the growth-suppressive effect through restoration of RASSF10 in QGY7703 and HepG2. We have established stable RASSF10-transfected cell lines and the ectopic expression of RASSF10 was confirmed by RT-PCR and western blot (Figure 2a). Restored expression of RASSF10 was observed in RASSF10-transfected cells in contrast with the vector-control (pcDNA3.1) cells. We also evaluated whether the artificially restored expressions of RASSF10 in HCC cell lines were obviously obviated from the natural level of RASSF10 in normal liver cells, which could result in extra but not natural biological functions for RASSF10 in liver cells. No excessively higher level of RASSF10 expression was found as compared with the normal liver control. The ectopic expression of RASSF10 in these HCC cell lines caused an inhibitory effect on cell viability (Figure 2b) and colony formation (Figure 2c). No significant difference in cell apoptosis drived by RASSF10 was observed by FITC Annexin V Apoptosis Detection assay (Figure 2d).

### Ectopic expression of RASSF10 suppresses HCC cells migration or invasion

To further gain the insights into the tumor suppressive property of RASSF10, we investigated the effect of RASSF10 on migration in QGY7703 and HepG2 Cells. Ectopic expression of RASSF10 distinctly inhibited HCC cells migration (*P*<0.01) (Figure 2e). The suppressive effect on HCC cells was further confirmed by invasion assay (*P*<0.05) (Figure 2f), suggesting RASSF10 could be involved in the mechanisms of HCC metastasis.

### Induction of cell cycle accumulation in G1 phase by RASSF10

**Figure 3:**
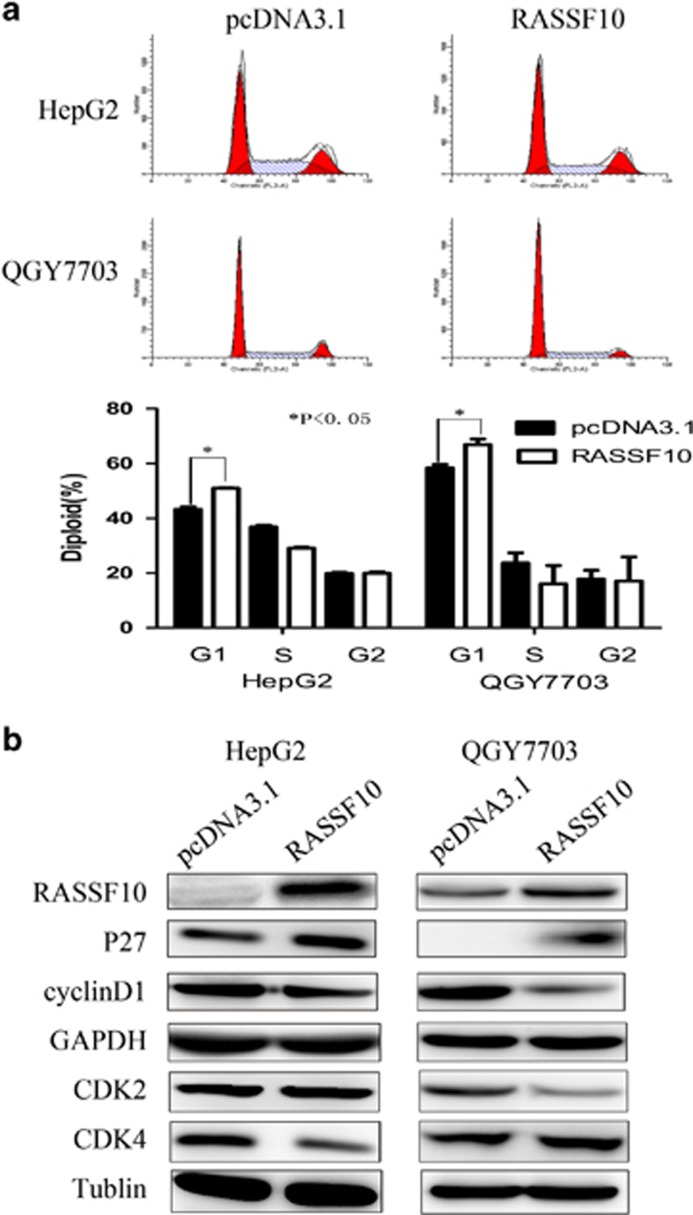
RASSF10 modulated cell cycle. (**a**) Cell-cycle distribution was analyzed by FACS flow cytometry in QGY7703 cells and HepG2 cells stably transfected with pcDNA3.1-RASSF10 or pcDNA3.1 vector. Restoration of RASSF10 induced the accumulation of HCC cells in G1 cell cycle phase. The asterisk indicates statistical significance (*P<0.05). (**b**) Western blot shows the expression of major mediators in cell cycle process including p27, CyclinD1, CDK2 and CDK4.

We evaluated the cell cycle modulation to the growth inhibition in HCC cells induced by RASSF10. The number of cells in G1 phase following RASSF10 restoration was substantially increased (50.96±0.20% vs control 43.28±0.95% in HepG2 cells; 67.92±0.95% vs control 59.37±0.95% in QGY7703 cells; *P*<0.05). Cell cycle modulation was further assessed by detection protein expression of P27, CyclinD1 and cyclin-dependent kinase (CDKs) using western blot in the QGY7703 and HepG2 cells (Figure 3b). Our results showed that re-expression of RASSF10 up-regulated the level of p27 and down-regulated cyclinD1 and CDK2/CDK4 expressions.

### RASSF10 inhibits tumor growth in nude mice.

**Figure 4:**
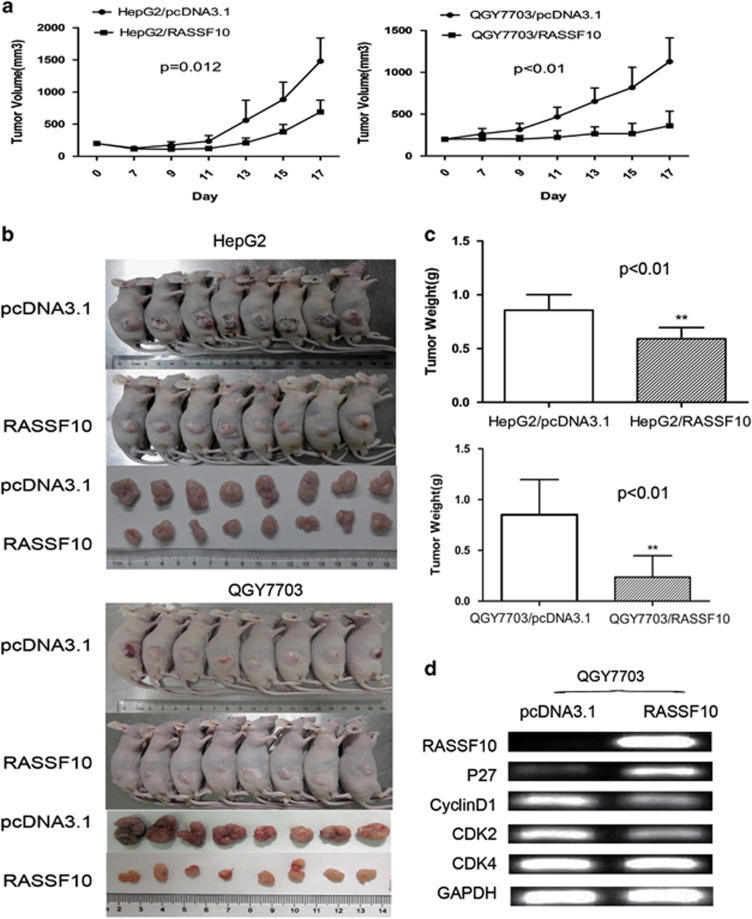
RASSF10 retarded tumor growth *in vivo*. (**a**) Subcutaneous tumor growth curve of RASSF10-expressing QGY7703 and HepG2 cells in nude mice was compared with vector (pcDNA3.1) transfected cells. The RASSF10 group showed a retarded tumor growth compared with the vector group (HepG2, *P*=0.012; OGY7703, *P*<0.01). The data are means±s.d. (*n*=8/group). (**b**) A representative picture of tumor growth in nude mice subcutaneously inoculated with RASSF10 or vector (*n*=8/group). (**c**) Histogram represents mean of the tumor weight from the RASSF10 and vector groups. The asterisk indicates statistical significance (*P<0.05, **P<0.01). (**d**) Cell cycle mediators including p27, Cycling D1, CDK2 and CDK4 were evaluated in the xenograft tumors by RT-PCR.

We subsequently investigated the effects of RASSF10 expression on the tumorigenic potential of HCC cells *in vivo*. QGY7703 and HepG2 cells stably transfected with RASSF10 or empty vector were injected subcutaneously into nude mice and the animals were closely monitored for tumor growth. The tumor volume was significantly lower in RASSF10-transfected nude mice as compared with the vector control mice (QGY7703, *P*<0.01; HepG2, *P*=0.012) (Figure 4a). At the end of experiments, tumors were isolated and weighed. The mean tumor weight was significantly less in RASSF10-transfected nude mice as compared with the vector control mice (*P*<0.01) (Figures 4b and c). Finally, the level of RASSF10 and cell cycle modulating genes including p27, cyclinD1, CDK2 and CDK4 were validated in the xenograft/QGY7703 tumors by RT-PCR. Up-regulation of RASSF10 increased the level of p27 and down-regulated cyclinD1 and CDK2/CDK4, which was consistently agreed with the results observed *in vitro* study (Figure 4d).

### MMP2 involved in the invasion suppression induced by RASSF10

**Figure 5:**
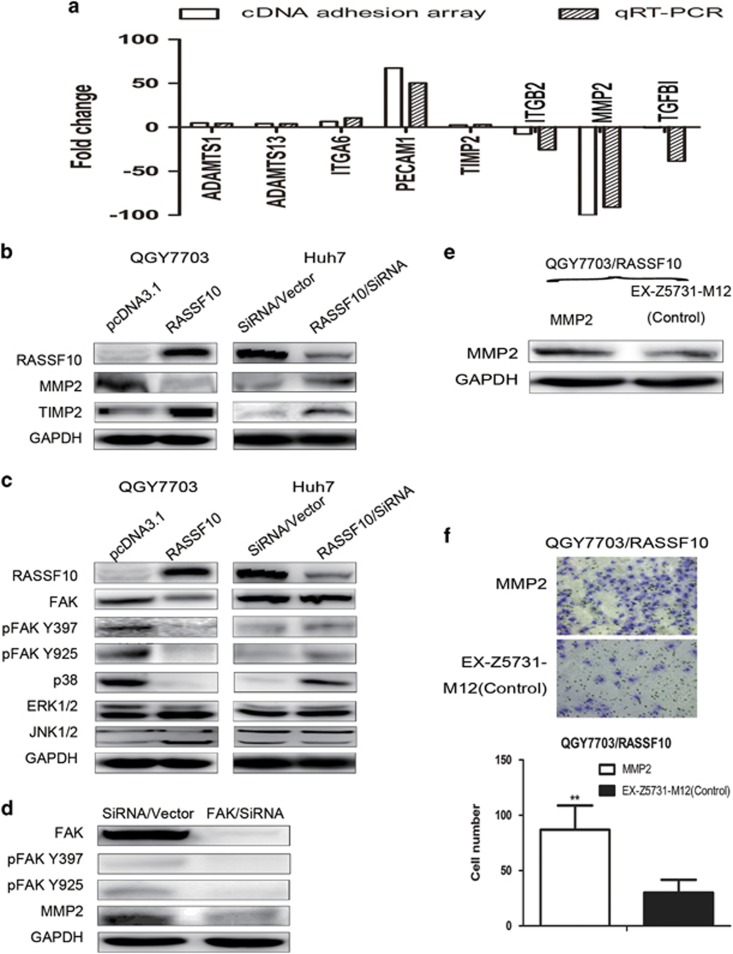
RASSF10 impaired cell adhesion through MMP2 via FAK signaling. (**a**) Eight genes modulated by RASSF10 in HCC migration or invasion were found by cDNA adhesion array, which were further confirmed by qRT-PCR. White bars indicate the result of cDNA adhesion array, and black bars represent qRT-PCR data (the value of 2^−ΔΔ^CT) in QGY 7703 transfected with pcDNA3.1/RASSF10 or empty vector (pcDNA3.1). (**b**) Western blot confirmed the association between RASSF10 and MMP2 or TIMP2 by over-expression or knock-down assay. (**c**) Regulatory effect of RASSF10 on FAK and MAPKs. Over-expression of RASSF10 has suppressed the accumulation of total or phosphorylation FAK and p38 MAPK; consistently, down-regulation of RASSF10 by SiRNA/RASSF10 in Huh 7 cells induced the activity of FAK, as well as p38 MAPK. The expression of ERK1/2 or JNK1/2 was independent on the level of RASSF10. (**d**) Regulatory effect of FAK on MMP2. Depletion of FAK suppressed the expression of MMP2. (**e**) Rescued assay for MMP2 in stable cell lines (QGY7703/RASSF10) was evidenced by western blot. (**f**) The change of cell invasion property was re-evaluated following MMP2 rescued assay. More invaded cells were observed with the restored expression of MMP2 in QGY7703/RASSF10. Invaded cells were stained with cell stain solution, counted by microscope in five random high power fields. Data are mean±s.d. The asterisk indicates statistical significance (*P<0.05, **P<0.01).

To identify the potential downstream targets modulated by RASSF10 in HCC migration or invasion, we performed the adhesion gene-expression array in QGY7703/RASSF10 cells. Ectopic over-expression of RASSF10 altered multiple adhesion associated genes expression (supplementary Table 1), including the increased levels of TIMP2, ADAMTS1, ADAMTS13, ITGA6 and PECAM1 (>2 fold change), and the down-regulation of MMP2, ITGB2 and TGFBI (<−2 fold change), which were further validated by qRT-PCR analysis (Figure 5a). TIMP2 is known as an endogenous MMP2 inhibitor.^25^ Re-expression of RASSF10 in QGY7703 cells was confirmed to significantly suppress MMP2 protein, and to induce the expression of TIMP2 by western blot (Figure 5b). Consistently, suppression of RASSF10 in Huh7 cells has increased the level of MMP2; however, TIMP2 expression was not down-regulated as expectation following the inhibition of RASSF10 (Figure 5b).

### Invasion suppression by RASSF10 is mediated by MMP2 through FAK signaling pathway

As the inconsistent corresponding between TIMP2 and MMP2 in varied HCC cells, we supposed any other modulating pathway may involve in the RASSF10-MMP2 expression model. Notably, FAK and MAPK pathway have been reported to be associated with the production of MMPs.^26, 27^ We verified the importance of these pathways under regulation by RASSF10 in HCC by over-expression or knock down assay. Re-expression of RASSF10 repressed total FAK and the active forms of FAK, phosphor-FAK Y397 (pFAK Y397) and phosphor-FAK Y925 (pFAK Y925), as well as p38 MAPK, but no significant changes in the expression of ERK1/2 and JNK1/2 (Figure 5c). In keeping with these results, transfection of RASSF10/siRNA has induced pFAK Y397, pFAK Y925 protein and p38 MAPK in Huh 7 cells (Figure 5c).

To further confirm the interaction between FAK and MMP2 in HCC, we examined the effects of FAK on MMP2 *in vitro* by knock down assay and western blot analysis. As shown in Figure 5d, protein expression of MMP2 was significantly decreased following the suppression of FAK in Huh 7 cells.

Finally, we investigated whether the RASSF10-associated-invasion-suppression was mediated by the inhibition of MMP2. The expression of MMP2 was restored in QGY7703/RASSF10 (Figure 5e) and the change of invasion characteristics was revaluated. Re-expression of MMP2 has facilitated the invasion of QGY7703/RASSF10, which has ever suppressed by the ectopic expression of RASSF10 (*P*<0.01) (Figure 5f).

## Discussion

HCC is the second common causes of cancer death worldwide with 745 000 deaths per year.^28^ Considerable efforts have been focused on unraveling novel tumor suppressor genes and molecular mechanisms to design better treatments to reduce its high mortality.

RASSF10 is the latest identified member of the RASSF family. In this study, we found RASSF10 was frequently silenced or down-regulated in HCC cell lines and HCC tissues as comparing with their adjacent non-tumor tissues, suggesting RASSF10 would be a potential tumor suppressor in the carcinogenesis of HCC. Hypermethylation of CpG islands were more prevalent in HCC cells lines than in normal control. The silenced expression of RASSF10 in those HCC cells was rescued by the pharmacological demethylation treatment, indicating DNA methylation could be one of the principal regulatory mechanisms for RASSF10 inactivation. Concordance with the latest report about RASSF10 in gastric carcinoma, we also observed mild methylation of RASSF10 in normal liver, but the expression of RASSF10 was not decreased.^29^ The normal tissue exhibited much weaker extent of methylation than those HCC cells, which showed down-regulated or silenced RASSF10 (Supplementary Figure 1a), indicating the inactivity of transcription induced by methylation could be associated with the extent of promoter methylation.

The putative tumor suppressor function of RASSF10 in liver cancer was verified by *in vitro* and *in vivo* assays. Transgenic expression of RASSF10 in two silenced HCC cell lines (QGY7703 and HepG2) showed significant growth-suppressive effect evidenced by cell viability and colony formation. The suppression of tumor growth was further confirmed by subcutaneous xenograft nude mice model. The accumulation of cells in G1 phase was revealed in both RASSF10-reexpressed-QGY7703 and −HepG2, which was mediated through the increase of p27, a CDK inhibitor,^30^ followed by the decrease of cyclin D1 or CDK2/4. Collectively, our findings indicate that RASSF10 functions as a tumor suppressor in hepatocarcinogenesis.

Metastasis is the other important property for malignant tumors. Some reports have showed the reduced expression of RASSF10 was significantly associated with the advanced tumor stage in prostate carcinoma and glioma.^18, 19^ Our *in vitro* study has showed RASSF10 significantly suppressed invasion or migration in HCC cell lines. However, we failed to confirm the decrease of RASSF10 was associated with the lymph nodes metastasis or tumor stage in the HCC patients. Higher ratio of patients was observed with down-regulated RASSF10 in N0 group than those in N1 group. The conflicting results may contribute to the limited cases we have included (only five cases for N1 group) in the study. Metastasis is a complex procedure, in which cancer cell should migrate away from the primary tumor and invade or degrade the surrounding extracellular matrix, which is mediated by a series of functional molecules such as collagens, lamininsand and fibronectin. MMPs are key proteins implicated in extracellular matrix remodeling and degradation by metastatic cells.^31^ MMP2 is a zinc-dependent endopeptidase involved in tumorigenesis, metastasis and angiogenesis through interacting with extracellular matrix.^32, 33, 34, 35, 36^ Suppression of MMP2 was reported to inhibit the invasion and metastasis of HCC *in vitro* and *in vivo*.^37^ RASSF10 was found to inhibit the expression of MMP2 in HCC cells. Furthermore, the inhibition of invasion by RASSF10 could be compensated by the rescued MMP2 expression in HCC cells, suggesting RASSF10 could interrupt invasion or migration of HCC cells through MMP2 suppression.

TIMP2 is known as an important protein performing an endogenous MMP2 inhibitor function.^25^ Over-expression of RASSF10 has facilitated the expression of TIMP2, speculating RASSF10 could mediate MMP2 by TIMP2. However, silence of RASSF10 by siRNA in Huh7 cells has not suppressed TIMP2 expression as suppose, suggesting the effect of RASSF10 on TIMP2 could be varied in different types of HCC cells. Some other signal pathways modulating MMP2 by RASSF10 could be involved in HCC cells, such as FAK or MAPK signaling pathway.^26, 27, 38^ FAK is a focal adhesion-associated protein kinase involved in cellular adhesion and spreading processes.^39^ FAK activity elicits intracellular signal transduction pathways that promote the cells contacting with the extracellular matrix and promoting cell migration. Activation of FAK leads to the secretion of MMP2;^40, 41^ conversely, silence of FAK has decreased the accumulation of MMP2 protein. Our study showed knock-down of RASSF10-activated FAK with increased phosphorylation of Tyr 397 (FAK/Src) and phosphorylate Tyr 925. Furthermore, over-expression of RASSF10 blocks the activation of FAK and then inhibits MMP2 expression.

Several reports have associated p38 MAPK signaling with the regulation of EMT.^27, 42, 43^ The expression of MMP-1, MMP-2, MMP-9 and MMP-13 has been shown to be mediated by p38α MAPK in the prostate, breast and liver cell lines derived from human tumors.^44, 45, 46^ Activation of p38α MAPK can trigger cell migration and cytoskeleton remodeling in tumor cells^42, 43^ and is required for the invasive capacity of pancreatic and hepatocellular carcinoma cell lines.^47, 48^ Here, the loss of p38 MAPK mediated by RASSF10 was supposed to involve in the inhibition of invasion or migration through MMP2 in HCC cells.

In conclusion, we identified RASSF10 as a novel tumor suppressor inactivated by promoter methylation in HCC. RASSF10 involves in the suppression of hepatocarcinogenesis by inhibiting cell growth and cell invasion through regulating MMP2 in the FAK signaling or p38 MAPK pathway.

## Materials and methods

### Ethics statement

Investigation has been conducted in accordance with the ethical standards, and according to both the declaration of Helsinki and national and international guidelines, and has been approved by the authors' institutional review board.

### Cell lines and tissue samples

The liver cancer cell lines including HepG2, Hep3B, Huh7, PLC-5, BEL7404, SK-Hep1, QGY7701 and QGY7703 were graciously gifted from Dr Jun Yu of Chinese University of Hong Kong, China, which have been authenticated in the lab before. Normal liver cell line LO2 was kindly gifted from Zhejiang Provincial Key Laboratory of Laparoscopic Technology, Hangzhou. Hepatic tumors from 2007 to 2014 were collected in Sir Run Run Shaw Hospital. Metastatic liver cancers were excluded in the study and total 52 paired liver tissues including primary HCCs and their adjacent normal tissues were included (*α*=0.05, *β*=0.2). The informed consent was obtained from each patient and the procedure was approved and supervised by authors' institutional review board.

### Reverse transcription-polymerase chain reaction (RT-PCR) and quantitative real-time PCR (qRT-PCR)

Total RNA and genomic DNA were extracted with Trizol reagent (Invitrogen, Carlsbad, CA, USA) following manufacturer's instruction. The concentrations were quantified by NanoDrop 2000 (Nanodrop, Wilmington, DE, USA). Reverse transcription reaction was performed using 1 μg of total RNA with Reverse Transcriptase M-MLV (Takara Bio, Dalian, China). The expression levels of RASSF10 were determined by RT-PCR with Taq polymerase (Takara Bio, Dalian, China) and quantitative real-time PCR (qRT-PCR) with SYBR Green Master Mix Kit (Takara Bio, Dalian, China) in an ABI 7500 PCR system (Cell Signaling Technology, Beverly, MA, USA). Glyceraldehyde-3-phosohate dehydrogenase (GAPDH) was used as an internal control. RT-PCR was 35 cycles with an annealing temperature of 56 °C and the expression levels of RASSF10 mRNA in tissues were determined using the 2^−∆ΔCt^ method. All primers sequences are listed in Supplementary Table 2.

Methylation-specific polymerase chain reaction and BGS DNA was bisulfite treated with Zymo DNA Modification Kit (Zymo Research, Orange, CA, USA) according to the protocol provided by the manufacturer. The bisulfite-modified DNA was amplified using primer pairs that specifically amplify either methylated (forward-5-TAGAGCGTAGTCGTAATCGC-3; reverse-5-CCGAAATCTACTAAAACGACG-3; 169 bp) or unmethylated (forward-5-GGTAGAGTGTAGTTGTAATTGT-3; reverse-5-AACCAAAATCTACTAAAACAACA-3; 173 bp) sequences of the RASSF10 promoter CpG islands. BGS was carried out for 45 cycles with annealing temperature at 55 °C. For BGS, PCR products amplified with BGS primers was subjected to DNA sequencing. The RASSF10-BGS primers are as follows: forward-5-TATTTTTAGTTATAGTTTTGGGTT-3 and reverse-5-ACCAACTTCTCTTCCTAACAA-3 (408 bp). 5-aza-2'-deoxycytidine (5-aza-DC) treatment cells (HepG2, Hep3B, BEL7404, QGY7701 and QGY7703) were treated for 72 h with 5 μM 5-aza-2'-deoxycytidine (5-aza-DC) (Sigma, St Louis, MO, USA) for induction of demethylation and then harvested for RT-PCR analysis. An equivalent concentration of the vehicle dimethyl sulfoxide (DMSO) was used as the control.

### Construction of expression vector and cell transfection

The RASSF10 expression vector was obtained from Chinese University of Hong Kong. The MMP2 expression vector was structured by Biosea Company (Biosea, Hangzhou, China). Constructs were confirmed by way of sequencing. Cells were cultured in 24-well plate for 12 h and transfected with pcDNA3.1-RASSF10 or empty vector pcDNA3.1 using Lipofectamine 2000 (Invitrogen, Carlsbad, CA, USA). Transfected cells (QGY7703 and HepG2) were selected by G418 (200 μg/ml, 300 μg/ml) to generate stable cell lines validated by RT-PCR and western blot. Stable cell lines (QGY7703/RASSF10) then were transfected with EX-Z5731-M12-MMP2 or empty vector EX-Z5731-M12 evidenced by western blot.

### Colony formation assay

Cells (QGY7703 and HepG2) transfected with pcDNA3.1-RASSF10 or pcDNA3.1 empty vector were selected with G418 for 10–14 days. The surviving cells were then stained with gentian violet after methanol fixation. Colonies with cell numbers of more than 50 cells per colony were counted. The experiments were repeated at least three times.

### Cell viability assay

Cell proliferation was detected with cell viability assay with 3-(4,5-dimethylthiazol-2-yl)-2,5-diphenyltetrazolium bromide (MTT) reagents (Promega, Madison, WI, USA). Stable transfected QGY7703 (3 × 10^3^/well) were plated into 96 well for 0, 24, 48 and 72 h and HepG2 (5 × 10^3^/well) for 0, 1, 3, 5 and 7 days. After incubation with CellTiter 96 Aqueous One Solution reagent for 2 h, the absorbance was measured at 490 nm according to the manufacture instruction. The experiments were repeated at least three times.

### Cell migration and invasion assay

QGY7703 and HepG2 cells transfected with pcDNA3.1 vector or pcDNA3.1-RASSF10 were used for cell migration and invasion assays. Cell migration was assessed by modified Boyden transwell chambers assay (Corning Costar; Cambridge, MA, USA). Briefly, 5 × 10^4^ cells/well were plated into 150 μl of 1% fatal bovine serum (FBS) medium in the upper chamber, and 600 μl of medium containing of 15% FBS were added to the lower chamber. The cells were incubated for 20 h. The non-migratory cells in the upper chamber were removed with a cotton swab. The cells on the bottom of the membrane were fixed and stained with 2-(4-amidinophenyl)-6-indolecarbamidine dihydrochloride (DAPI). The number of visible cells was counted by fluorescence microscope in five random high power fields.

To evaluate cell invasion, we use BD Matrigel Basement Membrane Matrix (final concentration of 200–400 μg/ml) (BD Biosciences, Franklin Lakes, NJ, USA) coating on transwell chambers. The cells were incubated for 24–48 h. Briefly, invaded cells on the bottom of the membrane were counted by microscope in five random high power fields or incubated with cell stain solution and transfected 100 μl of dye/solute mixture to a 96-well plate for colorimetric measurement at 560 nm. All the experiments were repeated three times.

### Flow cytometry

Cell cycle distribution was performed using the cell cycle staining kit (Multisciences Biotech, Hangzhou, China) by flow cytometry. Stably transfected cells (QGY7703 and HepG2) were collected and incubated with mixture (DNA staining solution and permeabilization solution) for 30 min. Cell cycle distribution were determined using the flow cytometry (Becton Dickinson, Mountain View, CA, USA) and were analyzed with Modify software (Phoenix, San Diego, CA, USA).

Cell apoptosis assays were performed using FITC Annexin V Apoptosis Detection Kit I (BD Biosciences) by flow cytometry analysis. Briefly, stably transfected cells (QGY7703 and HepG2) were stained with fluorescein isothiocyanate (FITC)-Annexin V and propidium iodide and were analyzed by FACScan flow cytometry. The experiments were repeated at least three times.

### *In vivo* tumorigenicity

Male ethylic nude mice (nu/nu) (4–5 weeks old) were purchased from Shanghai Laboratory Animal Co. Ltd (SLAC, Shanghai, China). According to the past experience, all the mice were randomly but averagely divided into four groups (marked as A, B, C and D group, *n*=8/group). Animal lab technician was blind to the detail information about each animal group. Stably transfected cells (QGY7703 or HepG2) (1 × 10^7^ cells in 0.2 ml BD matrigel basement membrane matrix diluted 1:1 with phosphate-buffered saline) or the corresponding control cells were injected subcutaneously into the dorsal right flank of mice. Tumor diameter was measured every 2 days for 2 weeks. Tumor volume (mm^3^) was estimated by measuring the longest and shortest diameter of the tumor and calculated as described.^49^ The mice were sacrificed and the tumors were weighed at the end of the experiments. All experimental procedures were approved by the Committee of Animal Ethics, Zhejiang University.

### Oligonucleotide transfection

RASSF10 SiRNA was purchased from genepharma (genepharma, Shanghai, China) and FAK SiRNA was purchased from Cell Signaling Technology (Cell Signaling Technology, Beverly, MA, USA). Cells (Huh7) were cultured in six-well plate for 12 h and transfected with RASSF10 siRNA or FAK siRNA using Lipofectamine RNAiMAX Reagent (Invitrogen, Carlsbad, CA, USA).

### The adhesion gene-expression array

Total RNA was extracted from stably transfected QGY7703 cells with pcDNA3.1-RASSF10 or pcDNA3.1 vector, and reverse transcribed to cDNA. The adhesion gene expression array was performed using TaqMan Adhesion Array 96-Well Plates (Life Technologies, Carlsbad, CA, USA) following the manufacturer's protocol in duplicate. The experiments were carried out two times. The data was performed using relative quantification (ddCt) study. We selected ratio ⩾2 or ⩽−2 as the threshold for up-regulation or down-regulation. Eleven representatives of target genes were verified with qRT-PCR. All primers for qPCR are listed in Supplementary Table 2.

### Western blot

Protein expression of candidate genes was evaluated by western blot. Primary antibodies used in this study are as follows: RASSF10 (1:1000, catalog number: ab113105), MMP2 (1:1000, catalog number: ab86607) and tissue inhibitor of metalloproteinases 2 (TIMP2) (1:200, catalog number: ab180630) (Abcam, Cambridge, MA, USA); cyclin-dependent kinases2 (CDK2) (1:200, catalog number: sc-748), CDK4 (1:200, catalog number: sc-260), Janus kinase 1/2 (JNK1/2) (1:200, catalog number: sc7345), p38-mitogen activated protein kinase (p38 MAPK) (1:200, catalog number: sc-4708) (Santa Cruz Biotechnology, Dallas, TX, USA); P27(1:1000, catalog number: #3686 s), CyclinD1 (1:1000, catalog number: #2978); extracellular regulated protein kinases (ERK) (1/1000, catalog number: #4695), FAK (1:1000, catalog number: #3285), pFAK397 (1:1000, catalog number: #3283 s) and pFAK 925 (1:1000, catalog number: #3284 s) (Cell Signaling Technology, Beverly, MA, USA); GAPDH (1:1000, catalog number: 85-14-9523-80) and Tublin (1:1000, catalog number: 85-41-4510-82) (Multisciences Biotech, Hangzhou, China). The blots were developed using a chemiluminescence with Las-4000 Imaging System (Fujifilm, Tokyo, Japan).

### Statistical analysis

Statistical analysis was carried out by the SPSS 16.0 statistical software SPSS 16 (SPSS Inc., Chicago, IL, USA). Student *t* test was used to compare the differences of RASSF10 expression on the effect of colony formation, cell proliferation, cell migration and invasion. Data were expressed as the mean±s.d. of at least three independent experiments. The difference in tumor growth rate between the two groups of nude mice was determined by repeated-measures analysis of variance. The differential RASSF10 mRNA expression between primary HCCs and adjacent normal tissues was skewed distribution and was analyzed by the Wilcoxon-matched pairs test. The two-side significant level was set at *P*<0.05 through the whole-analysis process.

## Abbreviations

5-Aza-DC: 5-aza-2'-deoxycytidine
BGS: bisulfitegenome sequencing
CDKs: cyclin-dependent kinases
cDNA: complementary DNA
ECM: extracellular matrix
ERK: extracellular signal regulated kinase
FAK: Focal Adhesion Kinase
GAPDH: glyceraldehyde-3-phosphate dehydrogenase
HCC: hepatocellular carcinoma
JAK: Januskinase
MMP2: Matrix Metalloproteinase 2
MSP: methylation-specific polymerase chain reaction
pFAK Y397: phosphor-FAK Y397
pFAK Y925: phosphor-FAK Y925
p38 MAPK: P38-mitogen activated protein kinase
RASSF: Ras-Association Domain Family
qRT-PCR: quantitative real-time PCR
RT-PCR: Reverse transcription-polymerase chain reaction
TIMP2: tissue inhibitor of metalloproteinases 2.
