# Supplementary figures and images for: Ras-association domain family 10 acts as a novel tumor suppressor through modulating MMP2 in hepatocarcinoma
Source: Oncogenesis. 2016 Jun 27;5(6):e237–. doi: 10.1038/oncsis.2016.24 (PMC4945738; doi:10.1038/oncsis.2016.24)

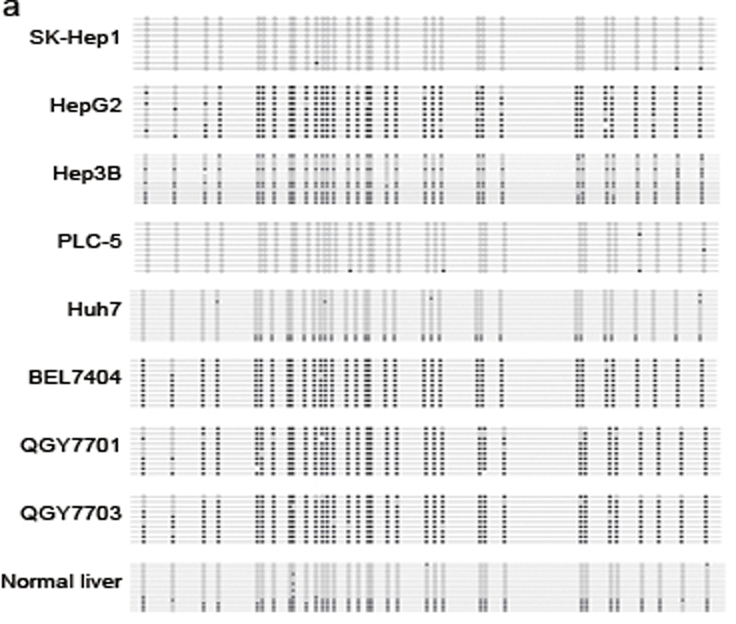

Supplement: Supplementary Figure 1 [file oncsis201624x1.tif]
