# Supplementary material for: Ras-association domain family 10 acts as a novel tumor suppressor through modulating MMP2 in hepatocarcinoma
Source: Oncogenesis. 2016 Jun 27;5(6):e237–. doi: 10.1038/oncsis.2016.24 (PMC4945738; doi:10.1038/oncsis.2016.24)
Supplement: Supplementary Table 1 [file oncsis201624x2.doc]

| **S-Table1** Representative gene expressionprofile in RASSF10 transfectants compared with vector control (fold change) by Taqman 96-well adhension array | | | | |
| --- | --- | --- | --- | --- |
| Gene | Description | Accession | Fold change | Gene Function* |
| B2M | Beta-2-Microglobulin | NM_004048.2 | 4.3845 | Im |
| ADAMTS1 | ADAM Metallopeptidase With Thrombospondin Type 1 Motif, 1 | NM_006988.3 | 4.964 | An,Inf |
| ADAMTS13 | ADAM Metallopeptidase With Thrombospondin Type 1 Motif, 13 | NM_139025.3 | 4.132 | Ad |
| COL12A1 | Collagen, Type XII, Alpha 1 | NM_004370.5 | 10.1345 | Ad |
| COL5A1 | Collagen, Type V, Alpha 1 | NM_000093.4 | 4.665 | Ad |
| ITGA6 | Integrin, Alpha 6 | NM_000210.2 | 6.6645 | Ad |
| LAMA2 | Laminin, Alpha 2 | NM_000426.3 | 4.3455 | Ad,M |
| LAMB3 | Laminin, Beta 3 | NM_000228.2 | 3.3725 | Ad,M |
| PECAM1 | Platelet/Endothelial Cell Adhesion Molecule 1 | NM_000442.4 | 67.475 | Ad,Ap,Inf,M |
| THBS3 | Thrombospondin 3 | NM_001252607.1 | 2.581 | Ad |
| TIMP2 | TIMP Metallopeptidase Inhibitor 2 | NM_003255.4 | 2.5455 | Ad,An,Inf,Inv,M |
| CLEC3B | C-Type Lectin Domain Family 3, Member B | NM_003278.2 | 4.8705 | Ad |
| VTN | Vitronectin | NM_000638.3 | 27.853 | Ad |
| ADAMTS8 | ADAM Metallopeptidase With Thrombospondin Type 1 Motif, 8 | NM_007037.4 | 0.000149 | An |
| COL6A1 | Collagen, Type VI, Alpha 1 | NM_001848.2 | 0.2125 | Ad |
| ITGA2 | Integrin, Alpha 2 | NM_002203.3 | 0.2965 | Ad |
| ITGB2 | Integrin, Beta 2 | NM_000211.3 | 0.133 | Ad |
| MMP2 | Matrix Metallopeptidase 2 | NM_001127891.1 | 0.01 | Ad,An,Inf,Inv,M |
| MMP9 | Matrix Metallopeptidase 9 | NM_004994.2 | 0.286 | M |
| SPARC | Secreted Protein, Acidic, Cysteine-Rich | NM_003118.3 | 0.0315 | Cg |
| TGFBI | Transforming Growth Factor, Beta-Induced | NM_000358.2 | 0.1985 | Ad |

*adhesion (Ad), angiogenesis (An), apoptosis (Ap) , cell growth (CG), immunity (Im),inflammation(Inf), invasion(Inv), migration (M)
