# Supplementary material for: Ras-association domain family 10 acts as a novel tumor suppressor through modulating MMP2 in hepatocarcinoma
Source: Oncogenesis. 2016 Jun 27;5(6):e237–. doi: 10.1038/oncsis.2016.24 (PMC4945738; doi:10.1038/oncsis.2016.24)
Supplement: Supplementary Table 2 [file oncsis201624x3.doc]

| Supplementary Table 2 Primer sequences used for quantitative real-time PCR or RT-PCR | | | |
| --- | --- | --- | --- |
| Gene | Primer | Sequence | PCR product(bp) |
| RASSF10 | Forward | 5'CTGGTGCATCTGGTGCTT3' | 101 |
|  | Reverse | 5'TGCACCTTGGCCTCGTA3' |  |
| RASSF10 | Forward | 5'GTTCAGCAGGAGGAGTTGCT3' | 318 |
|  | Reverse | 5'CGTCAGCTCCAAAGTGTGCAA3' |  |
| GAPDH | Forward | 5'GAAGGTGAAGGTCGGAGT3' | 226 |
|  | Reverse | 5'GAAGATGGTGATGGGATTTC3' |  |
| MMP2 | forward | 5'TGGCAAGGAGTACAACAGC3' | 174 |
|  | reverse | 5'TGGAAGCGGAATGGAAAC3' |  |
| MMP9 | forward | 5'GCAGCCCCTGGTCCTGGTGC3' | 210 |
|  | reverse | 5'AGAAGCAGCAGCGCAGGC3' |  |
| TIMP2 | forward | 5'GTTCAAAGGGCCTGAGAAGGA3' | 166 |
|  | reverse | 5'CCAGGGCACGATGAAGTCA3' |  |
| TGFBI | forward | 5'CATTGAGAACAGCTGCATCG3' | 255 |
|  | reverse | 5'AGTCTGCTCCGTTCTCTTGG3' |  |
| SPARC | forward | 5'CAAGAAGCCCTGCCTGATGAGA3' | 213 |
|  | reverse | 5'GGGGGTGTTGTTCTCATCCAGC3' |  |
| PECAM1 | forward | 5'AGAAAACCACTGCAGAGTACCAG3' | 77 |
|  | reverse | 5'GGCCTCTTTCTTGTCCAGTGT3' |  |
| ADAMTS8 | forward | 5'TCCTGACTGTGTCTGGTGAGGT3' | 113 |
|  | reverse | 5'GATGTTGGTGCTTGCTCTTTCTT3' |  |
| ITGA6 | forward | 5'TCCCTGAACCTAACGGAGTCT3' | 254 |
|  | reverse | 5'ATGTCCAAGTAGTTCAGTTTG3' |  |
| ITGB2 | forward | 5'CCCACAAGCCTCGCTGAAACC3' | 276 |
|  | reverse | 5'CACGAAGGACCCGAAGCCAAT3' |  |
| ADAMTS1 | forward | 5'AAGCTGCTGATGGCACATATATTCA3' | 195 |
|  | reverse | 5'TTTTAGGTCGAAGGGCATTGC3' |  |
| ADAMTS13 | forward | 5'ACCTGGAGCTGCCTCATTAC3' | 389 |
|  | reverse | 5'CACTTCTCCACGCCACATTC3' |  |
| P27 | forward | 5' GCA CAC TTG TAG GAT AAG TGA AATGG3' |  |
|  | reverse | 5' CCT ATT CTA CCC AAC ACA GCA TTT AC3' |  |
| CyclinD1 | forward | 5' CCCTCGGTGTCCTACTTCA3' |  |
|  | reverse | 5' CTCCTCGCACTTCTGTTCCT3' |  |
| CDK2 | forward | 5' GCCAGAAACAAGTTGACGG3' |  |
|  | reverse | 5' ATGAGGGGAAGAGGAATGC3' |  |
| CDK4 | forward | 5' CTGGACACTGAGAGGGCAAT3' |  |
|  | reverse | 5' GGGACAAGAGGGAACATACC3' |  |
